# Long‐term follow‐up of patients treated with laser balloon for atrial fibrillation: A high volume center experience with the first‐ and second‐generation laser balloon

**DOI:** 10.1002/joa3.13088

**Published:** 2024-06-14

**Authors:** Lukas Urbanek, Stefano Bordignon, Shota Tohoku, Jun Hirokami, Takahiko Nagase, Shaojie Chen, David Schaack, K. R. Julian Chun, Boris Schmidt

**Affiliations:** ^1^ Cardioangiologisches Centrum Bethanien Agaplesion Markus‐Krankenhaus Frankfurt/M Germany; ^2^ Department of Cardiology Sakakibara Heart Institute Fuchushi Tokyo Japan

**Keywords:** ablation, arrhythmia, atrial fibrillation, laser balloon

## Abstract

**Background:**

Laser balloon (LB) pulmonary vein isolation (PVI) is an established ablation technique for atrial fibrillation (AF). We report long‐term follow‐up and procedural data of LB‐PVI and we compare the first and second LB generation.

**Methods:**

Patients undergoing LB ablation with first‐ (LB1) or second‐generation LB (LB2) for AF were retrospectively enrolled and divided into two groups. Procedural endpoint was complete PVI. Clinical success was defined as no recurrence of AF/atrial tachycardia after a 90 days blanking period.

**Results:**

538 patients were included (age 66 ± 10 years, 58% paroxysmal AF), 427 in LB1 and 111 in LB2. 2079 PVs were targeted and 2073 (99.7%) were successfully isolated; 2027 (97.5%) using solely the LB. Additional touch‐up ablation was limited (46 PVs; 2.2%) with no difference between the groups. Procedural (LB1: 120 ± 33 minutes vs. LB2: 99 ± 22 min; *p* < .001) and fluoroscopy time (LB1: 11.2 ± 5 min vs. LB2: 8.5 ± 3 min; *p* < .001) were shorter with LB2. The complication rate was 8.9% (LB1: 10.1% vs. LB2: 4.5%; *p* = .067) with most complications resulting from the access site (21/48). Overall freedom from AF after 1‐year was 73.7% (paroxysmal AF: 76.9%; persistent AF: 69.3%; *p* < .001) with no difference between the groups (LB1: 73.4% vs. LB2: 74.7%; *p* = .491).

**Conclusion:**

LB showed a high efficacy and acceptable safety, with numerically lower complication rates with the second‐generation LB. Procedure and fluoroscopy times were shorter with LB2. Overall, 73.7% of patients were free from AF at 1‐year, with comparable results among both generations.

## Graphical abstract

We report the outcomes of our comparison between patients treated with the first‐ and second‐generation laser balloon (LB). Both generations have demonstrated high effectiveness, notably with significantly shorter procedure times observed with the second‐generation LB. Long‐term follow‐up showed no difference between the two generations for either paroxysmal or persistent atrial fibrillation.

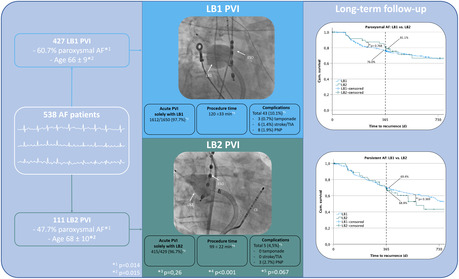

## 1 BACKGROUND

Pulmonary vein isolation (PVI) is the cornerstone for ablation of atrial fibrillation (AF).^1^ While point‐by‐point radio frequency (RF) ablation is the established gold standard for PVI, its technical complexity and steep learning curve have triggered the development of different ablation systems. Among these, the cryoballoon (CB) and the LB have emerged, demonstrating non‐inferiority to RF.^2^, ^3^, ^4^ The LB (LB, HeartLight‐LB1 or Excalibur‐LB2 HeartLight, CardioFocus, Marlborough, MA, USA) represents an innovative visually guided balloon approach. This technique enables direct visualization and ablation at the left atrial‐pulmonary vein (PV) junction.^5^ Randomized comparisons have underscored its noninferiority compared to RF with a similar 1‐year freedom from AF^3^, ^4^ and a high rate of acute and durable PVI.^6^

Concerning procedural time, LB1 has demonstrated similar procedure times as RF,^4^ albeit longer than those with CB.^7^ The optimized second‐generation LB (LB2, HeartLight Excalibur Balloon; CardioFocus) has introduced notable refinements, such as a more compliant balloon, resulting in an enhanced tissue contact and PV occlusion.^8^

Although diverse studies have individually outlined procedural characteristics and long‐term outcomes for LB1 and LB2, data on comparisons between LB1 and LB2 is scarce. In this study, we sought to analyze the procedural data, acute efficacy, and long‐term success in patients with symptomatic AF undergoing PVI with LB and compare the first‐ and second‐generation.

### 1.1 Patient population

We performed a retrospective clinical cohort study based on our institutional registry database. All patients with symptomatic AF (paroxysmal/persistent AF), who underwent ablation with LB1 or LB2 at Cardiologisches Centrum Bethanien Frankfurt a.M. were included. LB1 procedures were conducted from 06/2010 to 10/2017, whereas LB2 procedures were performed from 10/2017 to 04/2019. In both, LB1 and LB2, six operators were involved. The study protocol was approved by the institutional review board. The study complied with the Declaration of Helsinki.

Procedural data were recorded prospectively; follow‐up data were collected retrospectively. AF type was categorized as paroxysmal (lasting <1 week or episodes that are cardioverted within 7 days) and persistent (lasting >1 week and episodes that are terminated by cardioversion, either with drugs or by direct current cardioversion after 7 days or more).

### 1.2 Preprocedural care

Prior to the ablation procedure, a transesophageal echocardiography (TEE) was performed to rule out intracardiac thrombi in patients with AF. Patients in sinus rhythm (SR), who maintained a consistent anticoagulation regimen were exempt from undergoing TEE assessment. Patients were instructed to skip the morning dose of direct oral anticoagulants (DOAC) on the day of ablation. Those receiving vitamin K antagonists continued their anticoagulation therapy, with the objective of maintaining an international normalized ratio (INR) of 2–3.

### 1.3 Ablation procedure

All procedures were carried out under the administration of deep sedation. This was achieved through the administration of boluses of midazolam, fentanyl, and a continuous infusion of propofol, administrated by trained nurses, working under the direct supervision of cardiologists who possessed specialized expertise in airway management. In all patients, an esophageal temperature probe (SensiTherm, St. Jude Medical, Inc., St. Paul, MN, USA) was inserted to monitor luminal esophageal temperature. During the procedure, heparin administration followed immediately the transseptal puncture, aiming for an activated clotting time of 300 s. To be noticed, neither for LB1 nor for LB2 procedures, an ultrasound‐guided venous puncture was used during the study period. Vascular accesses were obtained traditionally with a Seldinger technique and following anatomical markers.

### 1.4 LB PV isolation

**FIGURE 1:**
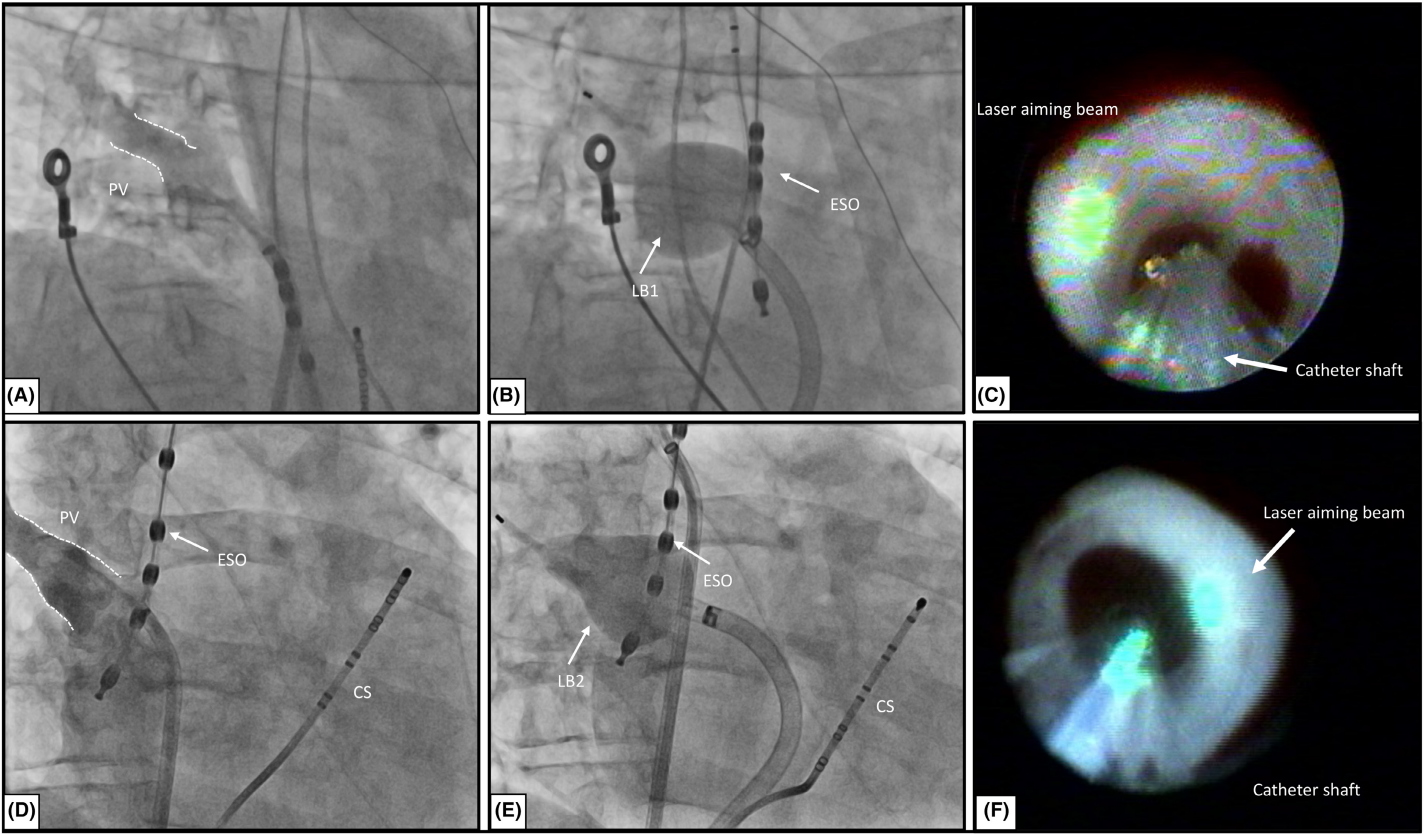
(A) Fluoroscopic view (RAO 30°) of a selective pulmonary vein angiography of the right superior pulmonary vein (RSPV). (B) Fluoroscopic view (RAO 30°) of the LB1 occlusion of the RPSV. (C) Endoscopic view of the LB1 positioned in the pulmonary vein (PV). The green aiming beam indicates the ablation spot (white arrow). (D) Fluoroscopic view (RAO 30°) of a selective pulmonary vein angiography of the RSPV. (E) Fluoroscopic view (RAO 30°) of the LB2 occlusion of the RSPV. (E) Endoscopic view of LB2) positioned in the pulmonary vein (PV). Note the better compliance of the second‐generation laser balloon (LB2). CS, coronary sinus catheter; ESO, esophageal probe; LB1, first‐generation laser balloon; LB2, second‐generation laser balloon; RSPV, right superior pulmonary vein.

A detailed description of how LB ablation is performed has been published previously.^9^ Subsequent to a single transseptal puncture facilitated by an SL1 sheath (Abbot medical) and a BRK‐1 needle (Abbot medical), selective PV angiograms were performed (Figure 1). Following the angiograms, the SL1 sheath was exchanged for the LB delivery sheath over a wire placed in the left superior PV.

The navigation of the LB to the individual PV was executed using a steerable sheath. Upon proper positioning, the balloon was inflated and gently pushed against the PV ostium to attain optimal PV occlusion. In patients with an LCPV, the balloon was maximally inflated to conform to the individual PV ostium, and both branches were encircled by a single circular lesion set. Validation of the ostial or antral position of the LB was accomplished by fluoroscopic criteria (Figure 1) or by the endoscopic image visualizing the equator marker placed proximal to the carina. The deployment of laser energy was executed in a point‐by‐point fashion, thereby covering 30° of a circle with each ablation lesion (Figure 1). The energy output was titrated according to the degree of tissue exposure between 5.5 and 12 W. Ablation energy was delivered for either 20 or 30 s, according to the manufacturer's presets. The operators were instructed to achieve a 30%–50% overlap among adjacent lesions to ensure comprehensive coverage. After complete visually guided circular ablation of all PVs, the catheter was exchanged to a 15 mm Spiral Catheter (Lasso‐Biosense Webster) to prove entrance block. In cases where residual left atrium to PV conduction persisted, additional ablation was strategically employed, using the LB based on the activation sequence indicated by the circular mapping catheter. During the applications at the septal PVs, the right phrenic nerve (PN) was constantly paced from the superior cava vein, and the PN function was monitored with palpation and with the compound motor action potential. In case of reduction of PN function (compound motor action potential decrease >30%), the application was immediately terminated. Phrenic nerve palsies (PNP) were defined as PN injury not resuming before discharge (<48H). In instances where the achievement of PVI using the balloon catheters proved to be unsuccessful, touch‐up ablation with the ThermoCool catheter (Biosense Webster, Inc., CA, USA) was performed under fluoroscopic guidance in combination with a lasso catheter placed in the targeted vein. The earliest site of activation was ablated.

### 1.5 Post‐procedural care

After ablation, pericardial effusion was ruled out with transthoracic echocardiography. Hemostasis was achieved through a figure‐of‐eight suture, followed by a 6‐h period of bed rest. In cases of prolonged bleeding, protamine antagonization and, if necessary, a mechanical compression device (FemoStop™, Abbott Laboratories) were used. DOACs were continued the evening of the ablation with half dose and were recommended for at least 2 months independent of the CHA2DS2‐VASc score. For two weeks, proton pump inhibitors were administered and antiarrhythmic drugs were stopped.

In case of AF recurrence within a 90 days blanking period, electrical or chemical cardioversion was performed and antiarrhythmic drugs were prescribed until the end of the blanking period.

### 1.6 Follow‐up

A 6‐ and 12‐month control including a 72‐hour Holter ECG was scheduled, and patients from external institutions could be followed up in their referral centers. Data from outpatient ward presentations, phone contact, and arrhythmia records from other hospitals and referring physicians were collected. Pacemaker interrogation was utilized when available. Chronic efficacy endpoints were defined as AF, atrial flutter, or atrial tachycardia exceeding 30 s after a 3‐month blanking period as well as the need for repeat ablation during the 3 months blanking period.

### 1.7 Statistical analysis

Quantitative values with normal distribution were presented as mean and standard deviation and compared by using the two‐tailed Student's *t*‐test. Ordinally and nominally scaled values were presented in absolute and percent frequencies. Contingency tables were used to test for association using the Pearson's chi‐squared test. Additionally, survival analyses according to Kaplan–Meier were performed and the log‐rank test was used to investigate AF‐free survival. To identify predictors of recurrence during follow‐up, we performed a univariate Cox regression. Thereafter, a multivariate analysis was performed using the variables with *p* < .05 in the univariate analysis to examine their independent associations.

The tests were performed two‐sided with a significance level of 5%. An alpha adjustment for multiple testing was not applied, and the results were interpreted accordingly in an exploratory and descriptive manner. Statistical calculations were done using the statistical programming software SPSS Statistics 26 (SPSS Inc. an IBM Company, Chicago, IL).

## 2 RESULTS

### 2.1 Patient baseline characteristics

Among the 538 enrolled patients, 427 underwent ablation with the first‐generation LB and 111 with the second generation. AF was paroxysmal in 312 (58%) and persistent in 226 (42%) patients. Most baseline characteristics were comparable among the groups (Table 1). The mean age was 66 ± 10 years. There were more patients with paroxysmal AF in LB1 compared to LB2 (60.7% vs. 47.7%; *p* = .014). There were also significantly more patients with heart failure in LB1 vs. LB2 (13.6% vs. 2.7%; *p* = .001). The mean CHA_2_DS_2_‐VASc‐Score was 2.4 ± 1.6. There was also no significant difference between the number of patients with PV anomalies, defined as common ostium of the left or the right PVs or a right middle PV (LB1: 16.2%; LB2: 12.6%; *p* = .357).

**TABLE 1:** Patient baseline characteristics.

Patient baseline characteristics	Entire cohort (*N* = 538)	LB1 (*N* = 427)	LB2 (*N* = 111)	*p*‐value
Age (years)	66 ± 10	66 ± 9	68 ± 10	.015
Gender (male) (%)	306 (56.9%)	243 (56.9%)	63 (56.8%)	.977
BMI (kg/m^2^)	28 ± 5	28 ± 6	28 ± 4	.350
Paroxysmal AF (%)	312 (58%)	259 (60.7%)	53 (47.7%)	.014
Hypertension (%)	380 (70.6)	305 (71.4)	75 (67.6)	.426
Coronary artery disease (CAD) (%)	105 (19.6)	81 (19.0)	24 (21.8)	.502
Heart failure (%)	61 (11.3)	58 (13.6)	3 (2.7)	.001
Diabetes mellitus (%)	64 (11.9)	48 (11.2)	16 (14.4)	.358
History of stroke or TIA (%)	34 (6.3)	29 (6.8)	5 (4.5)	.378
Left atrial diameter (mm)	40 ± 5	40 ± 5	41 ± 5	.546
Ejection fraction (%)	62 ± 7	62 ± 7	61 ± 6	.870
CHA_2_DS_2_VASc score	2.4 ± 1.6	2.4 ± 1.6	2.4 ± 1.4	.885
PV anomalies (%)	83 (15.4)	69 (16.2)	14 (12.6)	.357

Abbreviations: AF, atrial fibrillation; BMI, body mass index; CAD, coronary artery disease; LB1, first‐generation laser balloon; LB2, second‐generation laser balloon; PV, pulmonary vein; TIA, transient ischemic attack.

## 3 PROCEDURAL DATA

### 3.1 Procedural efficacy

**FIGURE 2:**
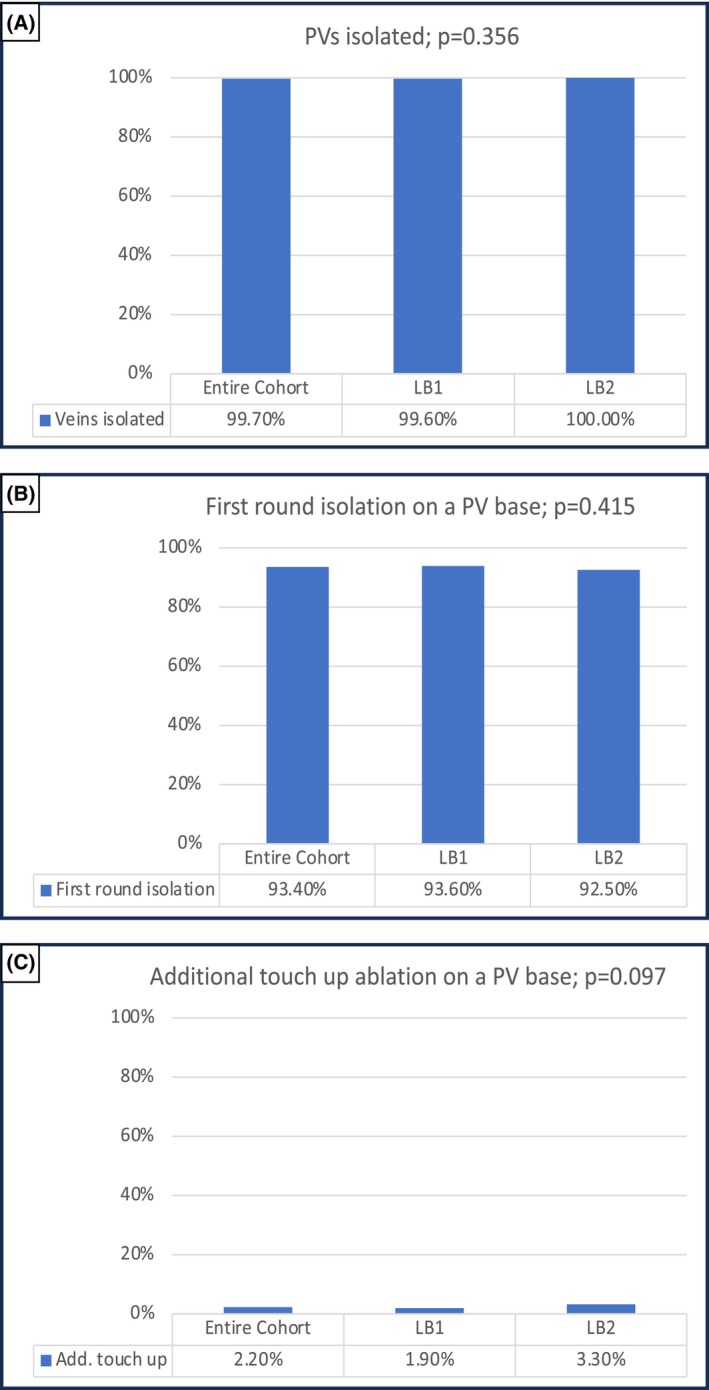
Comparison of procedural data between both laser balloon generations. (A) Rate of pulmonary veins isolated solely with the laser balloon in both techniques. (B) Comparison of first‐round isolations between LB1 and LB2. (C) Rate of additional touch‐up ablations for both laser balloon generations. LB1, first‐generation laser balloon; LB2, second‐generation laser balloon.

The procedural data is summarized in Tables 2, 3, 4 and Figure 2. Among the 538 patients, 2079 PVs were identified and 2073/2079 (99.7%; Figure 2A) were successfully isolated. In 500/538 (92.9%) patients and in 2027/2079 (97.5%) PVs (LB1: 97.7% vs. LB2: 96.7%; *p* = .26) PVI could be achieved using solely the LB. 52 PVs could not be isolated with the LB. Additional radiofrequency (RF) current touch‐up ablation was performed in 46 (2.2%) veins (LB1: 1.9% vs. LB2: 3.3%; *p* = .097; Figure 2C) or 33 (6.1%) patients (LB1: 5.2% vs. LB2: 9.9%; *p* = .063). In 20 veins touch‐up ablation was required because of failed LB PVI after several attempts, whereas in 26 it was necessary because of technical defects of the LB system. 6 veins in 5 patients (all in LB1) could not be isolated: one ablation was prematurely stopped because of a cardiac tamponade, another was aborted without complete PVI because of a PNP that impeded complete RSPV isolation. In three patients the RIPV could not be isolated despite several attempts. A high percentage of PVI with first encirclement by the LB (93.4%) was recorded for both generations (LB1: 93.6% vs. LB2: 92.5%; *p* = .415; Figure 2B and Table 3). On a PV base, the LCPV had the lowest rate of PVI with first encirclement (76.7%; Table 4) compared to the other veins (all >90%) and the need for touch up ablation was also higher in LCPVs (6.85%; Table 4).

**TABLE 2:** Procedural data on a patient basis.

	Entire cohort (*N* = 538)	LB1 (*N* = 427)	LB2 (*N* = 111)	*p*‐value
Procedural time (min)	116 ± 33	120 ± 33	99 ± 22	<.001
Fluoroscopy time (min)	10.7 ± 5	11.2 ± 5	8.5 ± 3	<.001
Additional touch‐up ablation RF (%)	33 (6.1)	22 (5.2)	11 (9.9)	.063
Complete PVI (%)	533 (99.1)	422 (98.8)	111 (100)	.589
CTI block (%)	24 (4.5)	16 (3.7)	8 (7.2)	.124

Abbreviations: CTI, cavotricuspid isthmus; LB1, first‐generation laser balloon; LB2, second‐generation laser balloon; PVI, pulmonary vein isolation; RF, radio frequency.

**TABLE 3:** Procedural data on a pulmonary vein basis.

	Entire cohort (*N* = 2079)	LB1 (*N* = 1650)	LB2 (*N* = 429)	*p*‐value
Isolation after first round	1942 (93.4%)	1545 (93.6%)	397 (92.5%)	.415
Additional touch‐up ablation	46 (2.2%)	32 (1.9%)	14 (3.3%)	.097
Complete PVI	2073 (99.7%)	1644 (99.6%)	429 (100%)	.356
Applications	28.1 ± 8	28.5 ± 8	26.5 ± 6	<.001

Abbreviations: CTI, cavotricuspid isthmus; LB1, first‐generation laser balloon; LB2, second‐generation laser balloon.

**TABLE 4:** Procedural data per pulmonary vein.

	Entire cohort	RSPV	RIPV	LSPV	LIPV	LCPV	RCPV	RMPV
Number of PVs	2079	532	532	465	465	73	6	6
Complete PVI (%)	99.7	99.8	99.3	100	100	98.6	100	100
Isolation after 1st round (%)	93.4	95.5	91.9	91.8	96.8	76.7	100	100
Additional touch‐up ablation (%)	2.2	1.1	3	2.8	1.3	6.85	0	0
Applications	28.1 ± 8	29 ± 7	25.7 ± 7	29.8 ± 7	25.5 ± 7	42.6 ± 12		

Abbreviations: LCPV, left common pulmonary vein; LIPV, left inferior pulmonary vein; LSPV, left superior pulmonary vein; PV, pulmonary vein; PVI, pulmonary vein isolation; RCPV, right common pulmonary vein; RMPV, right middle pulmonary vein; RIPV, right inferior pulmonary vein; RSPV, right superior pulmonary vein.

Procedure time (LB1: 120 ± 33 min; LB2: 99 ± 22 min; *p* < .001) and fluoroscopy time (LB1: 11.2 ± 5 min; LB2: 8.5 ± 3 min; *p* < .001) were significantly longer in LB1. The rate of concomitant CTI‐ablation (4.5%) was similar among the groups (Table 2). To be noted, only one esophageal injury was detected (symptomatic esophageal ulcera; LB1), most likely related to the ablation of the LIPV, which had normal diameters. The injury was treated conservatively and healed spontaneously. The average number of applications per vein was 28.1 ± 8 and was significantly higher in LB1 vs. LB2 (28.5 ± 8 vs. 26.5 ± 6; *p* < .001). The number of applications for each vein is summarized in Table 4 and was highest for the LCPV. A sub‐analysis of paroxysmal and persistent AF patients did not show any difference in procedural data.

### 3.2 Procedural safety

The overall (major and minor) complication rate was 8.9% (Table 5), with a numerical difference in favor of LB2 (LB1: 10.1% vs. LB2: 4.5%; *p* = .067). No periprocedural deaths were recorded. There were five strokes and one TIA in LB1 and none in LB2. Three of the five strokes reported postprocedural visual impairments after ablation, and only in one of these the symptoms were still present at the 3‐month follow‐up. One patient had sensory disturbances at his bottom lip after ablation that resolved spontaneously. One patient suffered from post‐procedural aphasia and hemiplegia of the left arm: a lysis therapy was initiated and the symptoms resolved without sequalae.

**TABLE 5:** Procedural safety.

Complications	Entire cohort (*N* = 538)	LB1 (*N* = 427)	LB2 (*N* = 111)	*p*‐value
TIA/Stroke	6 (1.1%)	6 (1.4%)	0	.354
Tamponade	3 (0.6%)	3 (0.7%)	0	1.0
Persistent phrenic nerve palsy	11 (2%)	8 (1.9%)	3 (2.7%)	.705
Complications at access site	21 (3.9%)	19 (4.4%)	2 (1.8%)	.275
Ventilation‐associated complications	1 (0.2%)	1 (0.2%)	0	1.0
Esophageal complications	1 (0.2%)	1 (0.2%)	0	1.0
Transient ST‐elevation	2 (0.4%)	2 (0.5%)	0	1.0
Pneumothorax	1 (0.2%)	1 (0.2%)	0	1.0
Hydrope decompensation	1 (0.2%)	1 (0.2%)	0	1.0
Pulmonary vein stenosis	1 (0.2%)	1 (0.2%)	0	1.0
Total	48 (8.9%)	43 (10.1%)	5 (4.5%)	.067

Abbreviations: LB1, first‐generation laser balloon; LB2, second‐generation laser balloon; TIA, transient ischemic attack.

Only one ventilation‐associated problem was detected in a patient requiring ventilation with a laryngeal mask. In total, 11 patients experienced a PNP (LB1: 1.9% vs. LB2: 2.7%; *p* = .705). Among these patients, 3 (27.3%) were asymptomatic and refused fluoroscopic controls. 4/11 patients showed a normal nerve function after 1 year. One patient was controlled for the first time after 3 years showing a normal nerve function and 3 (27.3%) patients still had a (partial) PNP after 3 months but refused further controls.

Three procedures were complicated by cardiac tamponade, all in LB1. In two cases the effusion developed slowly and was noticed after the isolation of all PVs. One ablation had to be stopped prematurely because of the tamponade. All three tamponades were managed with pericardiocentesis without cardiac surgery. One of the patients developed Dressler's syndrome, which was treated with non‐steroidal anti‐inflammatory drugs.

In total, there were 21 (3.9%) groin complications requiring intervention with no significant difference between the groups (LB1: 4.4% vs. LB2: 1.8%; *p* = .275). Most of them were groin false aneurysms that were treated with thrombin injection, others were lager haematoma that required blood transfusion. Due to a sick sinus node, 9 patients (all in LB1) required a temporary pacemaker, during/after the procedure, in 5 of these patients a permanent pacemaker was implanted.

### 3.3 Overall ablation outcome

**FIGURE 3:**
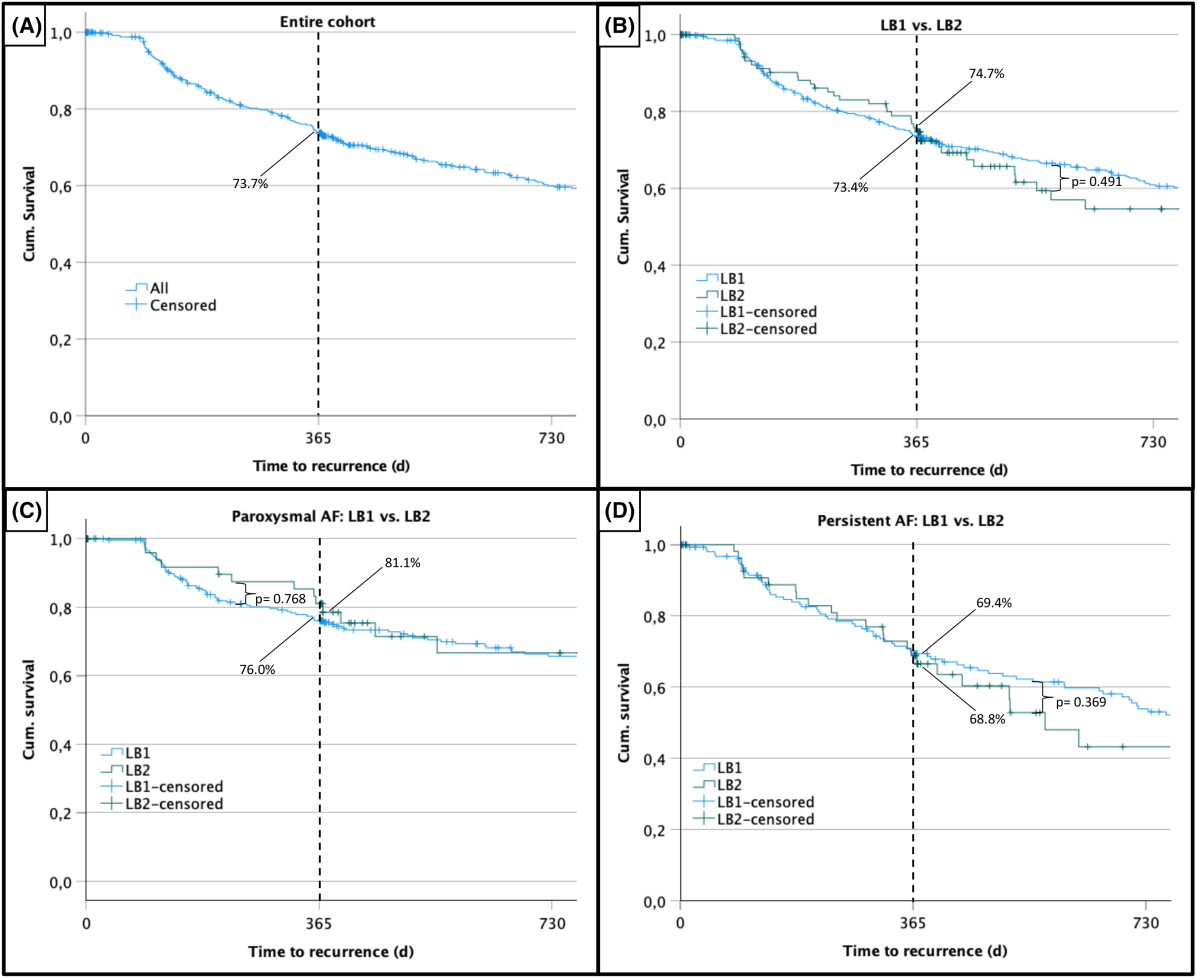
Kaplan–Meier survival analysis. (A) AF‐free survival of the entire cohort. (B) Comparison of AF‐free survival between LB1 and LB2. (C) Comparison of AF survival of LB1 and LB2 in paroxysmal AF. (D) Comparison of AF‐free survival of LB1 and LB2 in persistent AF. LB1, first‐generation laser balloon; LB2, second‐generation laser balloon.

Median follow‐up was 400 (186–1190) days. In total, there were 166 (30.9%) recurrences in blanking time with no difference between both LB generations (LB1: 31.4% vs. LB2: 28.8%; *p* = .604). The overall freedom from AF after 1 year was 73.7% (Figure 3A). The 1‐year freedom from AF in LB1 was 73.4% vs. 74.7% in LB2 (*p* = .491; Figure 3B). Furthermore, there was no difference in 1‐year freedom from AF between both LB generations for paroxysmal AF (LB1: 76% vs. LB2: 81.1%; *p* = .768; Figure 3C) and persistent AF (LB1: 69.4% vs. LB2: 68.8%; *p* = .369; Figure 3D). Moreover, age, recurrence in blanking, and persistent AF were found to be independent predictors of AF recurrence (Table 6). An additional multivariate analysis focusing solely on preprocedural parameters found age and persistent AF to be independent predictors of AF recurrence, while left atrial diameter slightly missed significance (*p* = .05).

**TABLE 6:** Uni‐and multivariate analysis for predictors of recurrences.

	Univariate analysis			Multivariate analysis			Multivariate analysis: Preprocedural parameters			
	HR	95% CI	*p*‐value	HR	95% CI	*p*‐value	HR		95% CI	*p*‐value
Age	1.036	1.021**–**1.051	<.001	1.039	1.017	1.061	<.001	1.043	1.021–1.065	<.001
Female gender	1.106	0.861**–**1.421	.429							
BMI	1.019	0.983**–**1.056	.298							
Persistent AF	1.630	1.270**–**2.093	<.001	1.368	1.038	1.803	.026	1.456	1.108–1.914	.007
LB 2	1.132	0.795**–**1.612	.491							
Hypertension	1.449	1.077**–**1.948	.014	1.157	0.790	1.695	.454	1.204	0.825–1.756	.336
Diabetes	0.824	0.548**–**1.237	.350							
Stroke	1.008	0.607–1.674	.974							
CHA₂DS₂‐VASc Score	1.123	1.041**–**1.212	.003	0.942	0.825	1.076	.381	0.935	0.820–1.067	.320
Heart failure	0.978	0.681**–**1.405	.906							
Coronary Artery Disease	1.060	0.772**–**1.453	.720							
Left atrial diameter (mm)	1.035	1.010–1.061	.006	1.021	0.994	1.049	.127	1.027	1.000–1.055	.05
Procedure time	0.999	0.995**–**1.003	.675							
Fluoroscopy time	1.007	0.984**–**1.030	.571							
PV‐Anomaly	0.792	0.558**–**1.125	.193							
Patients with single shot isolation of all veins	0.900	0.664–1.220	.497							
Recurrence in blanking	1.984	1.543–2.550	<.001	1.799	1.373	2.357	<.001			

Abbreviations: AF, atrial fibrillation; BMI, body mass index; CI, confidence interval; HR, hazard ratio; LB2, second‐generation laser balloon; PV, pulmonary vein.

## 4 DISCUSSION

We present a large retrospective analysis of patients treated with LB‐PVI for AF. Main findings are: (1) First‐ and second‐generation LB are both effective for PVI with 97.5% of PVs successfully isolated using solely the LB, (2) A high percentage of PVI with first encirclement (93.4%) for both generations (LB1: 93.6% vs. LB2: 92.5%), (3) LB ablation showed an acceptable safety profile, with an overall complication rate of 8.9% and a numerical trend to lower complication rates with LB2 (LB1: 10.1% vs. LB2: 4.5%; *p* = .067), (4) An overall 1‐year success of 73.7%; of which 76.9% in paroxysmal and 69.3% in persistent AF (*p* < .001).

### 4.1 Procedural efficacy and safety

In the presented cohort, we found a very high procedural acute success using both generations of LB. The need for touch up ablation was very limited with 2.2% (46 veins) for both generations (LB1: 1.9% vs. LB2: 3.3%; *p* = .097), which is in line with other LB studies^6^, ^10^ but more than the 0.3% that we reported with the cryoballoon.^11^

Moreover, we report a high percentage of PVI with first encirclement by the LB (93.4%), which is in line with the results of a meta‐analysis by Reynolds et al, reporting a rate of 89.3% of veins with first‐pass isolation.^12^ In our practice, we perform no preprocedural imaging and screening. Considering the lower rate of PVI with the first encirclement and the higher rates of touch‐up ablation for LCPV on a per‐vein basis, RF ablation should be considered in patients where an LCPV is identified in preoperative imaging. The procedural data, such as shorter procedural and fluoroscopy time, favoring LB2, may reflect the increased compliance of balloon‐facilitated occlusion of the targeted PV, along with a probable learning curve effect. The average number of applications was 28.1 ± 8 per PV, higher in LB1 vs. LB2 (28.5 ± 8 vs. 26.5 ± 6; *p* < .001). To be noticed, operators in our center are trained to use the highest energy dose allowed by the antrum visualization, and a high‐dose LB protocol (>8.5 W) was used in most of the applications. The overall complication rate was 8.9% with a trend to more complications in LB1 compared to LB2 (10.1% vs. LB2: 4.5%; *p* = .067). The overall similar safety profile of the two generations could be expected since the 2 LB generations share a similar architecture. Interestingly, all neurological complications were recorded using the LB1. The recorded longer procedure time and a learning curve effect could be associated with an increase in neurological complications. However, the better antral visualization, allowed by the more compliant LB2, may also reduce inadvertent laser delivery on blood and therefore lead to less clotting formation. Three cardiac tamponades occurred in LB1. In all procedures the operators described difficult transeptal puncture. 11 patients (2%) experienced a PNP, which is in line with other studies.^12^ Nearly half of the recorded complications (21/48; 44%) were access site complications, with a numerical trend to more complications in LB1. The reason is difficult to explain since the same operators were involved: once again, a learning curve effect cannot be excluded. To be noticed, echo‐guided venous puncture was not used in our series. The relatively high rate of patients with sick sinus syndrome in our cohort is likely linked to patient selection. Many of these patients had persistent AF, so sinus node dysfunction may not have been apparent beforehand. During the procedure, if a pause or bradycardia occurred after establishing SR, pacing was performed using the CS catheter. If necessary, a temporary pacemaker was subsequently implanted. In case of failed recovery in some cases, a permanent pacemaker was implanted thereafter.

### 4.2 Ablation outcome

The overall freedom from AF after 1 year was 73.7%. There was no significant difference in 1‐year freedom from AF between both LBs (LB1: 73.4% vs. LB2: 74.7%; *p* = .491). The similar results between the two generations could be interpreted as the results of the laser energy itself, which once deployed to the tissues assures a defined lesion durability. So, the introduction of the second‐generation LB2 with an improved visualization of target tissue simply improved how the operator is deploying the laser energy to the tissue, as summarized by the improved procedural parameters. A meta‐analysis reported a pooled estimate for 12‐month success for all AF types combined of 72.9% and among groups with only paroxysmal AF of 74.3%.^12^

A significant difference between paroxysmal and persistent AF in our cohort was found in LB1 (PAF: 76% vs. persistent AF: 69.4%; *p* = .001), but slightly missed significance in LB2 (PAF: 81.1% vs. persistent AF: 68.8%; *p* = .054), most likely because of a patient number not high enough to reach significance. The difference in follow‐up between PAF and persistent AF was also reported in other studies.^11^, ^13^

Our multivariate logistic Cox regression for predictors of recurrence showed that persistent AF was a predictor, as also reported elsewhere.^14^ Moreover, age and recurrence in blanking were predictors for AF recurrence. Especially recurrence during the blanking period was found in different studies^15^, ^16^ as a predictor for recurrence after blanking.

### 4.3 Limitations

This is a single‐center retrospective study of patients ablated with LB. Follow‐up data were mostly based on serial 24–72 h Holter ECG recordings and outpatient visits including 12‐lead ECG. As no implantable loop recorder was used, an overestimation of the chronic success rate is probable. A learning curve effect impacting the favorable data of LB2 cannot be excluded; however, most procedures were performed by experienced operators out of the learning curve, even with LB1. Ultrasound‐guided vascular puncture was not performed during this study period, probably explaining the high rate of vascular complications in this collective. The third generation of the LB (HeartLightX3) was not included in this analysis; nevertheless, the current data may be used as a comparator for the results obtained with the new technological improvement.

### 4.4 Conclusions

LB PVI with both generations is feasible with a high acute efficacy and acceptable safety profile. Overall, we observed 73.7% of patients with freedom from AF at 1 year, with no difference between both laser balloon generations. However, procedure time and fluoroscopy time were significantly shortened with the introduction of the second‐generation laser balloon.

## FUNDING INFORMATION

None.

## CONFLICT OF INTEREST STATEMENT

BS, SB, and KRJC have received speaking honoraria from Medtronic, Biosense Webster and Boston Scientific. SC has been invited as consultant to Boston Scientific and Biosense Webster.

## PATIENT CONSENT STATEMENT

Patients gave written informed consent to the procedure and data collection.

## ETHICS STATEMENT

The study was approved by the institutional review board and complied with the Declaration of Helsinki.

## Data Availability

The data underlying this article will be shared on reasonable request to the corresponding author.

## References

[1] Hindricks G , Potpara T , Dagres N , Arbelo E , Bax JJ , Blomstrom‐Lundqvist C , et al. 2020 ESC guidelines for the diagnosis and management of atrial fibrillation developed in collaboration with the European Association of Cardio‐Thoracic Surgery (EACTS). Eur Heart J. 2020:373–498. 10.1093/eurheartj/ehaa612 32860505

[2] Kuck K‐H , Brugada J , Fürnkranz A , Metzner A , Ouyang F , Chun KRJ , et al. Cryoballoon or radiofrequency ablation for paroxysmal atrial fibrillation. N Engl J Med. 2016;374(23):2235–2245. 27042964 10.1056/NEJMoa1602014

[3] Dukkipati SR , Cuoco F , Kutinsky I , Aryana A , Bahnson TD , Lakkireddy D , et al. Pulmonary vein isolation using the visually guided laser balloon: a prospective, multicenter, and randomized comparison to standard radiofrequency ablation. J Am Coll Cardiol. 2015;66(12):1350–1360. 26383722 10.1016/j.jacc.2015.07.036

[4] Schmidt B , Neuzil P , Luik A , Osca Asensi J , Schrickel JW , Deneke T , et al. Laser balloon or wide‐area circumferential irrigated radiofrequency ablation for persistent atrial fibrillation: a multicenter prospective randomized study. Circ Arrhythm Electrophysiol. 2017;10(12):e005767. 10.1161/CIRCEP.117.005767 29217521

[5] Reddy VY , Neuzil P , Themistoclakis S , Danik SB , Bonso A , Rossillo A , et al. Visually‐guided balloon catheter ablation of atrial fibrillation: experimental feasibility and first‐in‐human multicenter clinical outcome. Circulation. 2009;120(1):12–20. 19546385 10.1161/CIRCULATIONAHA.108.840587

[6] Dukkipati SR , Neuzil P , Kautzner J , Petru J , Wichterle D , Skoda J , et al. The durability of pulmonary vein isolation using the visually guided laser balloon catheter: multicenter results of pulmonary vein remapping studies. Heart Rhythm. 2012;9(6):919–925. 22293143 10.1016/j.hrthm.2012.01.019

[7] Chun JKR , Bordignon S , Last J , Mayer L , Tohoku S , Zanchi S , et al. Cryoballoon versus Laserballoon: insights from the first prospective randomized balloon trial in catheter ablation of atrial fibrillation. Circ Arrhythm Electrophysiol. 2021;14(2):e009294. 33417476 10.1161/CIRCEP.120.009294

[8] Nagase T , Bordignon S , Perrotta L , Bologna F , Tsianakas N , Chen S , et al. Analysis of procedural data of pulmonary vein isolation for atrial fibrillation with the second‐generation laser balloon. Pacing Clin Electrophysiol. 2019;42(7):837–845. 30969431 10.1111/pace.13692

[9] Bordignon S , Chun KR , Gunawardene M , Fuernkranz A , Urban V , Schulte‐Hahn B , et al. Comparison of balloon catheter ablation technologies for pulmonary vein isolation: the laser versus cryo study. J Cardiovasc Electrophysiol. 2013;24(9):987–994. 23800359 10.1111/jce.12192

[10] Rovaris G , Ciconte G , Schiavone M , Mitacchione G , Gasperetti A , Piazzi E , et al. Second‐generation laser balloon ablation for the treatment of atrial fibrillation assessed by continuous rhythm monitoring: the LIGHT‐AF study. Europace. 2021;23(9):1380–1390. 33837418 10.1093/europace/euab085

[11] Bordignon S , Chen S , Bologna F , Thohoku S , Urbanek L , Willems F , et al. Optimizing cryoballoon pulmonary vein isolation: lessons from >1000 procedures‐ the Frankfurt approach. Europace. 2021;23:868–877. 33458770 10.1093/europace/euaa406

[12] Reynolds MR , Zheng Q , Doros G . Laser balloon ablation for AF: a systematic review and meta‐analysis. J Cardiovasc Electrophysiol. 2018;29(10):1363–1370. 30016008 10.1111/jce.13698

[13] Valles E , Jimenez J , Marti‐Almor J , Toquero J , Ormaetxe JM , Barrera A , et al. Cryoballoon ablation for persistent and paroxysmal atrial fibrillation: procedural differences and results from the Spanish registry (RECABA). J Clin Med. 2022;11(5):1166. 10.3390/jcm11051166 PMC8910954 35268259

[14] Balk EM , Garlitski AC , Alsheikh‐Ali AA , Terasawa T , Chung M , Ip S . Predictors of atrial fibrillation recurrence after radiofrequency catheter ablation: a systematic review. J Cardiovasc Electrophysiol. 2010;21(11):1208–1216. 20487117 10.1111/j.1540-8167.2010.01798.x

[15] Kim YG , Boo KY , Choi JI , Choi YY , Choi HY , Roh SY , et al. Early recurrence is reliable predictor of late recurrence after radiofrequency catheter ablation of atrial fibrillation. JACC Clin Electrophysiol. 2021;7(3):343–351. 33516711 10.1016/j.jacep.2020.09.029

[16] Silva MR , Silva GS , Fernandes S , Almeida J , Fonseca P , Oliveira M , et al. Clinical relevance of the blanking period on late recurrence after catheter ablation of atrial fibrillation. J Cardiovasc Electrophysiol. 2022;34:24–34. 36317466 10.1111/jce.15729

